# Clinical and imaging associations for non-ketotic hyperglycemic chorea: a case-control study

**DOI:** 10.3389/fendo.2023.1323942

**Published:** 2023-12-20

**Authors:** Zhuo-Yuan Liang, Zhi-Hao Lu, Jian-Feng Qu, Yang-Kun Chen

**Affiliations:** ^1^ Department of Neurology, The Tenth Affiliated Hospital of Southern Medical University (Dongguan People’s Hospital), Dongguan, Guangdong, China; ^2^ Intelligent Brain Imaging and Brain Function Laboratory (Dongguan Key Laboratory), Dongguan People’s Hospital, Dongguan, Guangdong, China

**Keywords:** non-ketotic hyperglycemic chorea, diabetes mellitus, case-control study, renal dysfunction, association

## Abstract

**Background:**

The non-ketotic hyperglycemic chorea (NKHC) was a rare complication for patients with diabetes mellitus, but not been well studied. In the present research, we aimed to investigate the clinical and imaging characteristics of NKHC and explore the potential association.

**Methods:**

We performed a case-control study with patients diagnosed as NKHC. The patients with group of NKHC were retrospectively recruited, while the matched group were set to screened patients with diabetes mellitus but no NKHC at a 1:3 ratio. The clinical and imaging data were collected for all the participants of the two groups. Firstly, Correlation analysis was conducted to test the difference of all the variables between the NKHC group and matched group. Then, the putative associated factors for NKHC were further identified.

**Results:**

Eleven men and 9 women with NKHC and 60 matched participants were analyzed. The mean age of the NKHC group was 68.5 ± 14.9 years. Participants with NKHC were more likely to have a higher glycosylated hemoglobin (HbA1c) level (13 ± 2.82 vs. 10.57 ± 2.71, *P*<0.001), and a higher frequency of renal dysfunction (estimated glomerular filtration rates <60 ml/min/1.73m^2^) (55% vs. 20%, *P*=0.005). Logistic regression analyses showed that both higher HbA1c and renal dysfunction were significantly correlated with NKHC.

**Conclusion:**

A higher value of HbA1c and renal dysfunction may be associated with the occurrence of NKHC.

## Introduction

Chorea is a rare complication of diabetes mellitus reported more frequently in older Asian women (1) and was first described by Bedwell (2). Yahikozawa et al. suggested that a combination of choreatic movements and striatal hyperintensity on T1weighted MRI in diabetic patients may constitute a unique syndrome (3). Non-ketotic hyperglycemic chorea (NKHC), which manifests as a bilateral or unilateral chorea with hyperdensive lesions on non-enhanced computed tomography (CT) and hyperintensive lesions on T1-weighted magnetic resonance imaging (MRI) (4). Patients with NKHC show continuous, non-rhythmic, and involuntary movement of their limbs, that commonly persists for 1–2 weeks, or may last several months or years (1, 5). This movement disorder causes severe impairments in their activities of daily living. The prevalence of NKHC is underestimated with the rate of 1/100,000 due to the unfamiliarity and misdiagnosed by physicians (6).

Actually, previous study demonstrated that chorea, hyperglycemia, neuroimaging abnormalities in basal ganglia were “triple signs” for NKHC (7, 8). Sporadic case reports and literature reviews have suggested that genetic background, an age-related susceptibility, a sex predisposition, and glucose metabolism disorders might be involved in the development of NKHC (1, 5, 9). Rapid depleted GABA and acetylcholine in non-ketotic hyperglycemia cause a basal ganglia dysfunction and subsequent chorea (5). Reversible hyperintensive lesions on T1-weighted MRI may be the result of regional hypoperfusion, selective neuronal loss, gliosis and reactive astrocytosis, rather than cerebral infarction (10–12). Biopsy and post-mortem pathological studies have revealed that lesions of basal ganglia contained gliotic brain tissue, abundant gemistocytes, and hyalinosis of blood vessels (7). Necrosis, thickening of arteriole walls, narrowed vessel lumens, extravasation of red blood cells, capillary proliferation, lymphocytic infiltration, and macrophage invasion were also notable (10). However, most of the previous studies were case reports that only described the clinical and imaging manifestations of the disease (4, 13, 14). The associations between the clinical and neuroimaging factors for the NKHC have not been well characterized. In some case reports, patients with NKHC did not show a high level of blood glucose on admission (5). Most notably, the elevated glycosylated hemoglobin (HbA1c) was observed in some cases (5, 8). Therefore, we aimed to investigate the putative clinical and imaging characteristics of NKHC.

## Methods

### Research design and participants

We retrospectively recruited patients with NKHC who were admitted to the Department of Neurology or the Department of Endocrinology in the Tenth Affiliated Hospital of Southern Medical University (Dongguan People’s Hospital) between January 1^st^, 2012, and July 1^st^, 2023. The clinical definition of NKHC was according to Chua et al. (8), including: 1) the presence of bilateral or unilateral choreatic movements, 2) a history of diabetes mellitus, or a diagnosis of diabetes mellitus according to the 1999 World Health Organization criteria (15) although with normal blood glucose on admission, 3) absence of ketonemia, 4) hyperdensive lesions on brain CT or hyperintensive lesions on T1-MRI, what are mainly located on the unilateral/bilateral basal ganglia. Meanwhile, patients did not comply with the possible differential diagnoses of chorea combining to diabetes mellitus, along with wiping out the other possible diseases with basal ganglia neuroimaging abnormal findings. The inclusion criteria for the present study were (1) age >18 years; (2) be accordant to the criteria of clinical definition of NKHC; (3) the presence of bilateral or unilateral choreatic movements; and (4) complete data both of clinical and brain imaging (Both CT and MRI) records. The exclusion criteria were (1) acute stroke (including ischemic or hemorrhagic stroke) within the preceding 2 weeks; (2) the presence of a severe comorbidity (e.g., liver, kidney, heart, or respiratory failure; traumatic brain injury; or malignant tumor); (3) the presence of other movement disorders (e.g., Parkinson’s disease, Huntington’s disease, neuroacanthocytosis, paroxysmal kinesigenic dyskinesia or other choreatic diseases); (4) the presence of other metabolic disorders (e.g., hypocalcemia, and thiamine or nutritional deficiencies) and (5) the use of medication(e.g., dopaminergic medication, dopamine blocking agents, dopamine depleting agents, anti-seizure medications, calcium channel blockers, central nervous system stimulants, tricyclic, antihistamines, antidepressants and isoniazid) that has the potential to induce chorea-like symptoms during the preceding 2 weeks.

A control group of patients with diabetes mellitus but without NKHC were was carefully selected to match the NKHC at a ratio of 1:3. The members of this group were admitted to our hospital during the same period of time ( ± 3 months) and were carefully matched for sex, age ( ± 2 years) and admission blood glucose concentration ( ± 1 mmol/L). To be included in the non-NKHC group, patients had to meet the following criteria: (1) age >18 years; (2) diagnosis of diabetes mellitus made according to the 1999 World Health Organization criteria (14) and no diabetic ketoacidosis; (3) availability of complete medical and brain imaging (CT or MRI) records; and (4) the absence of movement disorders. The exclusion criteria were the same as for the NKHC group.

### Clinical data collection

We collected demographic and clinical information, including age, sex, history of diabetes mellitus (the disease duration, drug use, and self-monitoring of blood glucose) and the results of laboratory testing (e.g., blood glucose concentration on admission, HbA1c, liver enzyme activities, serum creatinine, and serum lipid concentrations). The self-monitoring of blood glucose (SMBG) was defined as: glucose monitoring was performed at least once per day in patients treated with insulin or at least three times per week in patients treated with oral agents or diet lonely. Renal function was assessed by estimated glomerular filtration rate (eGFR) was calculated with CKD-EPI equation (16). Renal dysfunction was defined as: eGFR < 60 ml/min/1.73m^2^.

### Neuroimaging measurements

All the participants in the two groups had data of brain CT scanning (Brilliance, Philips, Amsterdam, Netherlands) and MRI examination using a 1.5-T (Achieva, Philips) or a 3.0-T (Sonata, Siemens Medical, Erlangen, Germany) system within 48 hours of admission. The basic MRI sequences including: T1-weighted imaging (T1WI), T2-weighted imaging (T2WI), and magnetic resonance angiography (MRA). In addition, some of the participants also had the susceptibility-weighted imaging (SWI). Two neurologists (ZYL and JFQ), who were blinded to the participants’ clinical information, measured the CT and MRI parameters simultaneously, and inconsistencies in the data were resolved by discussion. We recorded the lesions located in the basal ganglia: including in the putamen, caudate nucleus and globus pallidus (8); which included hyperdense areas on CT and abnormal signals on each sequence of MRI. Because the basal ganglia are supplied by the middle cerebral artery (MCA), we also recorded the degree of stenosis in bilateral MCA, respectively. We also assessed any pre-existing abnormalities on brain MRI images as follows.

(1) White matter lesions (WMLs). The severity of WMLs was graded using the four-point scale described by Fazekas et al. (17). The WMLs included periventricular hyperintensities (PVHs) and deep white matter hyperintensities (DWMHs), which were separately scored on FLAIR images.
(2) Global brain atrophy. This was assessed using the ventricular-brain ratio (VBR) (18) on axial TI-weighted images. The width of the bodies of the lateral ventricles were measured half-way between the anterior and posterior limits (a), and the width of the brain was measured at the same level (b). The VBR was then calculated by dividing (a) by (b).
(3) Medial temporal lobe atrophy (MTLA). MTLA was evaluated using Schelten’s scale (19). The investigator judged the severity of MTLA on standard coronal MRI sections using a 0–4 range, with 0 representing no atrophy and 4 representing severe atrophy.
(4) Middle cerebral artery stenosis (MCA-S). MCA-S was defined as a signal loss of ≥50% and a reduction in the size of the arterial lumen or occlusion in the M1 segment of the MCA, identified using both the targeted maximal intensity projection on MRA and the source images to minimize the overestimation of stenosis that is inherent in the time-of-flight MRA technique (20).
(5) Enlarged perivascular spaces (EPVSs). According to the Standards for Reporting Vascular Changes on Euroimaging (STRIVE) guidelines, EPVSs are defined as fluid-filled spaces with signal intensities similar to those of cerebrospinal fluid on all of the sequences; that follow the course of penetrating vessels; are linear, round, or ovoid in shape; do not have a hyper-intense rim, to distinguish them from small lacunae; and generally have a diameter <3 mm (21) EPVSs can be detected as punctate or linear hyper-intensities on T2-weighted MRI, particularly in the basal ganglia (BG) and centrum semiovale (CS) (21, 22). EPVSs of small diameter (<3 mm) are often numerous and therefore impractical to count. In the present study, the severities of EPVS formation in the CS (CS-EPVS) and BG (BG-EPVS) were rated according to the number of spaces in the unilateral slice containing the largest number of EPVSs: score 0 = no EPVSs; 1 = 1–10; 2 = 11–20; 3 = 21–40; and 4 ≥40 (22).
(6) Silent brain infarcts (SBIs). SBIs were defined as cerebral infarcts that were evident on brain MRI and were not accompanied by overt clinical manifestations, such as rapidly developing clinical symptoms or signs of a focal loss of brain function (23). Most SBI are lacunae, which are visible as focal lesions with approximately the same intensity as cerebrospinal fluid on MRI and have diameters >3mm (24).

### Statistical analysis

The continuous variables with normal distributions were present as means and standard deviations, while the variables with skewed distributions were presented as medians and interquartile ranges. And the categorical variables were presented as frequencies and percentages. The normality of variables was tested with a one-sample Kolmogorov-Smirnov test. Comparisons of demographic and clinical variables were conducted between the NKHC group and matched group. We set up a logistic regression model step by step to find the putative associated factors of the clinical and imaging variables for NKHC. Firstly, univariate analyses were performed to compare the variables between the two groups to screened out the factors with significant difference, with a value of *P <*0.05. Secondary, trying to reduce the statistic bias, correlation analyses were conducted to test the collinearity of candidate independent variables. When the correlation coefficient between any of these putative factors was ≥ 0.40, then the variable with a lager statistic (with a lower P value) was determine to be included in the next step (25). Thirdly, the logistic regression analyses using a backward stepwise selection strategy were set up to study the putative correlation between these variables and NKHC. The odds ratio (OR) for each independent factor was calculated to compare its strength of the association for NKHC, when all the other factors were held constant. In addition, Spearman correlation analysis was conducted to test the factors that may affecting the resolution time in NKHC group. The significance level was set at 0.05 (two-sided), and statistical analyses were performed using the SPSS 27.0 statistical package (IBM Inc., Armonk, NY, USA).

## Results

### The characteristics of the participants with NKHC

**Figure 1:**
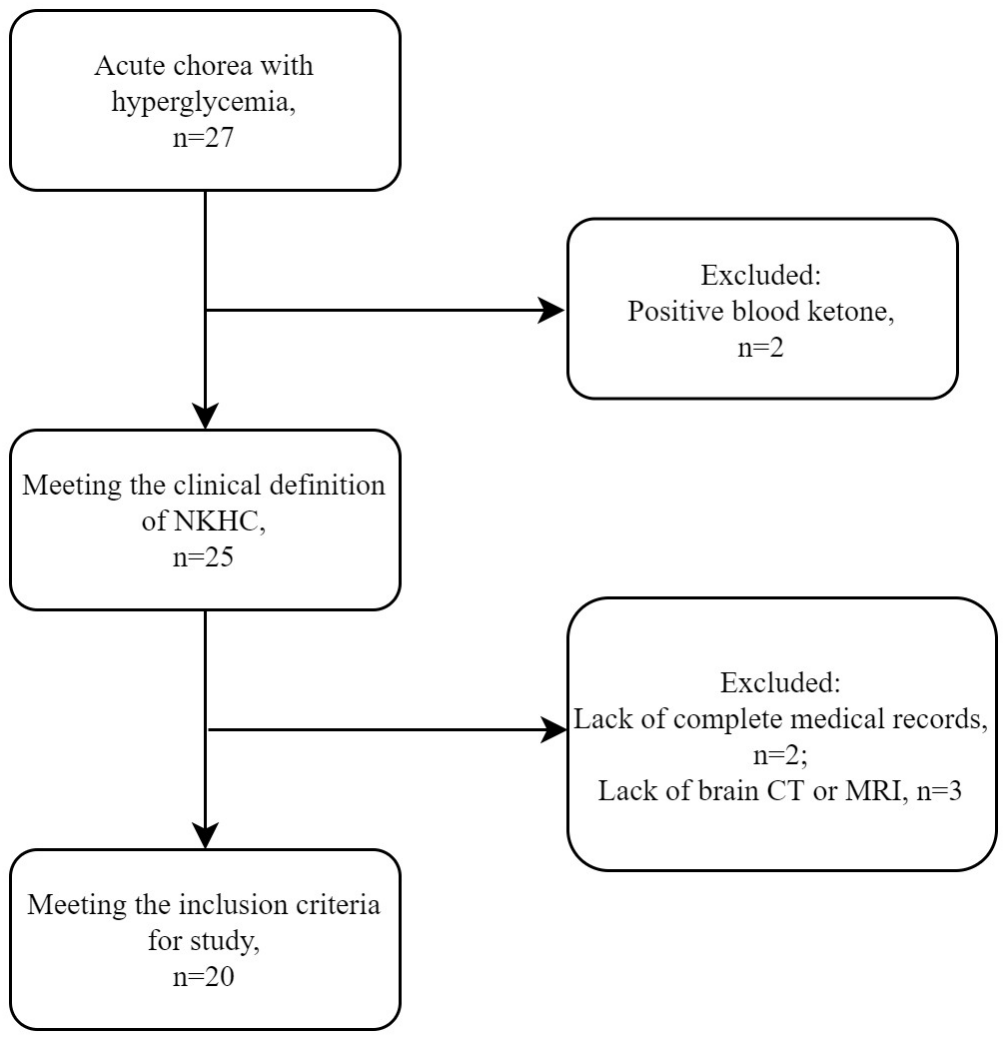
Selection of patients with NKHC for the present study. MRI, magnetic resonance imaging; CT, computed tomography; NKHC, non-ketotic hyperglycemic chorea.

During the study period, a total of 27 patients (14 female and 13 male) presented with acute or subacute chorea in the setting of hyperglycemia or diabetes mellitus. However, neuroimaging examination was not available for some patients, which precluded confident diagnosis of NKHC. Ultimately, 20 patients (9 female and 11 male) were included in the analysis, as shown in the flow chart (**Figure 1**). **Supplementary Table 1** compares excluded and included patients, while **Table 1** summarizes individual data for each NKHC participant. In the NKHC group, 45% were female, with a mean age of 68.5 ± 14.9 years. The median blood glucose concentration on admission was 23.1(11.35, 30.58) mmol/L, and the mean HbA1c was 13 ± 2.82%.

**Table 1:** Summary of the clinical features of the 20 participants with non-ketotic hyperglycemia chorea.

Case	Age	Sex	DM duration (yr)	BGCA (mmol/L)	HbA_1c_ (%)	Involved site	Hyperdensity on CT	Hyperintensity on T1-MRI	Hypointensity on SWI-MRI	Resolution time (day)	Additional anti-epileptic/anti-psychotic agents
1	75	F	8	32.8	12.6	face, LUE, LLE	R:PM,CN	R:PM,CN	ND	30	HPD,CZP,TP,AL
2	95	F	DOA	20.3	11.4	face, LUE, LLE, trunk	R:PM,CN,GP	R:PM,CN	ND	9	CZP
3	37	M	10	11.2	7.1	LUE	Negative	R:PM	ND	48	CBZ
4	57	M	DOA	36.6	15.4	LUE	Negative	R:PM	ND	12	CBZ
5	50	M	DOA	10.9	18.3	LUE,LLE	R:PM	R:PM	ND	19	CZP
6	67	F	20	5.9	11.6	LUE,LLE	Negative	R:PM,CN	ND	7	HPD,CZP
7	59	F	10	7.1	16.7	BE	B:PM,CN,GP	B:PM	B:PM,GP	25	HPD,CZP
8	73	M	30	32.6	14.6	LUE,LLE	R:PM,CN	R:PM,CN	ND	15	HPD,RPD
9	87	F	3	18.9	10.4	face, RUE, RLE	L:PM,CN	L:PM,CN	L:PM,CN	17	HPD,CZP
10	56	F	20	29.6	12.9	face, LUE, LLE	Negative	L:PM,CN	ND	20	HPD,CZP
11	54	M	DOA	22	13.3	LUE,LLE	R:PM,CN,GP	R:PM,CN,GP	R:GP	21	HPD,CZP
12	84	M	32	26.9	14.4	RUE,RLE	L:PM,CN,GP	L:PM,CN	L:PM	21	HPD,CZP
13	84	F	10	23.5	13.5	LUE	Negative	R:PM	B:GP	5	None
14	73	M	2	28.3	16.2	face, LUE, trunk	B:PM,CN,GP	B:CN	B:PM,CN,GP	7	None
15	84	F	20	11.8	13.8	LUE,LLE	R:PM	R:PM	B:PM,CN,GP	22	HPD
16	55	F	7	22.7	12.4	face, RUE, RLE	L:PM,CN	L:PM,CN	L:PM	25	HPD,CZP
17	83	M	DOA	26.7	15.7	RUE,RLE	L:PM,CN	L:PM,CN	L:CN,B:PM,GP	14	RPD
18	70	M	5	30.9	11.9	RUE	Negative	L:PM	B:GP	30	CZP
19	74	M	DOA	34.2	11.1	LUE, trunk	Negative	R:PM	ND	13	None
20	52	M	DOA	7.12	7.6	head, neck	Negative	B:CN	Negative	70	CZP

DM, diabetes mellitus; BGCA, blood glucose concentration on admission; DOA, diagnosis of diabetes mellitus on admission; L, left; R, right; B, bilateral; UE, upper extremity; LE, lower extremity; BE, bilateral extremities; PM, putamen; CN, caudate nucleus; GP, globus pallidus; ND, not done; HPD, haloperidol; CZP, clonazepam; CBZ, carbamazepine; AL, alprazolam; TP, tiapride; RPD, risperidone.

**Figure 2:**
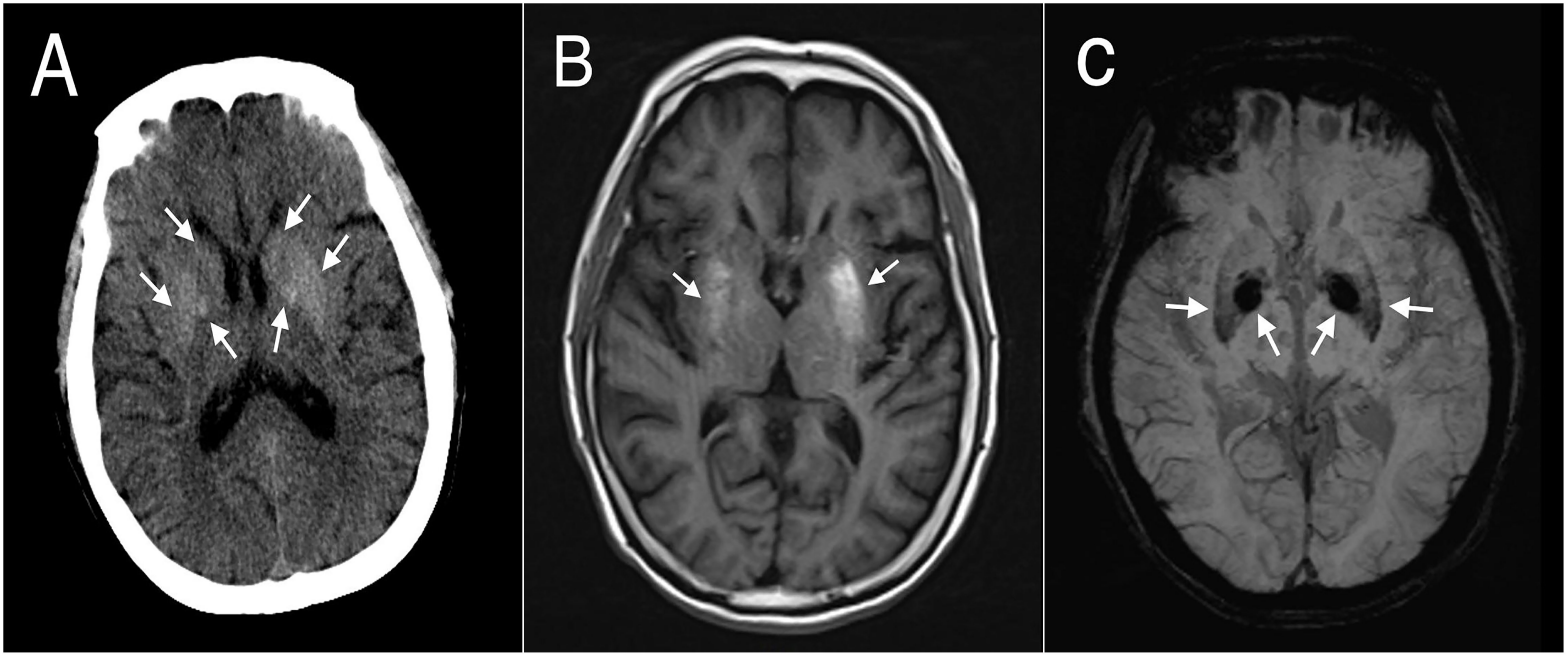
Brain computed tomography (CT) and magnetic resonance images (MRIs) of participant #7. **(A)** CT showed bilateral hyperdense lesions in the striatum. **(B)** T1-weighted MRI showed asymmetrical bilateral hyperintense lesions in the putamen, but no significant changes in the caudate or globus pallidus. **(C)** Susceptibility-weighted imaging showed bilateral symmetrical hypointense lesions in the putamen and globus pallidus.

One of the 20 participants with NKHC (No.7) involving their extremities bilaterally showed bilateral hyperdensive signals in their basal ganglia on CT, hyperintensive lesions on T1-weighted MRI, and hypointensive lesions on SWI (**Figure 2**). Five participants (No. 13, 14, 15, 17 and 18) with unilateral involvement of their extremities showed bilateral hypointensive lesions on SWI.

### Comparison of the clinical and imaging variable between the two groups

The results of the univariate analysis are presented in **Table 2**. Compared to the matched group, participants with NKHC had a higher frequency of irregular usage of glucose-lowering agents or no treatment (75% vs. 46.7%, *P*=0.038), a higher HbA1c level (13 ± 2.82% vs. 10.57 ± 2.71%, P<0.001), and worse renal function (55% vs. 20%, *P*=0.005).

**Table 2:** Comparison of the clinical characteristics and brain imaging variable between the NKHC group and matched group.

	NKCH N=20	Matched N=60	t/χ^2^/z-value	*P*-value
Clinical variables				
Age, years^*^	68.45 ± 15.3	68.18 ± 14.9	0.069	0.945
Sex, Female^†^	9(45%)	27(45%)	0	1
Prior Stroke^†^	7(35%)	9(15%)	3.75	0.102
Hypertension^†^	12(60%)	37(61.7%)	0.018	1
Dyslipidemia^†^	4(20%)	25(41.7%)	3.047	0.109
Coronary Artery Disease^†^	1(5%)	3(5%)	0	1
Duration of DM, years^‡^	6(0, 17.5)	7(2, 12.5)	-0.430	0.667
Irregular usage of glucose-lowering agents ^†^	15(75%)	28(46.7%)	4.844	**0.038**
No SMBG^†^	18(90%)	44(73.3%)	2.389	0.214
BGCA, mmol/L^‡^	23.1(11.35, 30.58)	23.05(11.5, 29.9)	-0.056	0.956
HbA_1c_, %^*^	13 ± 2.82	10.57 ± 2.71	3.504	**<0.001**
eGFR<60ml/min/1.73m^2‡^	11(55%)	12 (20%)	8.97	**0.005**
ALT, U/L^‡^	18.3 (12.3, 32.4)	18.6 (12.4, 30.4)	-0.072	0.942
TC, mmol/L^*^	4.78 ± 0.9	5.36 ± 1.49	-1.642	0.105
Imaging variables				
MCA-S^†^	8 (40%)	21 (35%)	0.162	0.790
DWMH^‡^	1 (0, 2)	1 (1, 2)	-0.323	0.746
PVH^‡^	1 (1, 2)	1 (1, 2)	-0.736	0.461
MTLA^‡^	2 (1, 2.75)	2 (1, 2.75)	-1.002	0.316
VBR^*^	0.21 ± 0.032	0.21 ± 0.045	0.501	0.618
EPVS (BG)^‡^	1(1, 2)	1(1, 2)	-1.089	0.276
EPVS (CS)^‡^	1(0, 1)	1(0.25, 2)	-1.675	0.094
SBIs^‡^	0(0, 2)	0.5(0, 2)	-0.574	0.566

^*^Mean (SD), t-test; ^†^ n (%), Fisher’s exact test; ^‡^ Median (interquartile range), Mann–Whitney U test; SMBG, self-monitoring of blood glucose; BGCA, blood glucose concentration on admission; eGFR, Estimated Glomerular Filtration Rate; ALT, serum alanine aminotransferase activity; TCH, serum total cholesterol concentration; Hyper-CT, hyperdense lesions on computed tomography; Hyper-TIWI, hyperintense lesions on the T1 sequence; Hypo-SWI, hypointense lesions on the SWI sequence; MCA-S, middle cerebral artery stenosis; DWMH, deep white matter hyperintensities; PVH, periventricular hyperintensities; MTLA, medial temporal lobe atrophy; VBR, ventricle/brain ratio; EPVS (BG), enlarged perivascular spaces in the basal ganglia; EPVS (CS), enlarged perivascular spaces in the centrum semiovale; SBIs, silent brain infarcts; NKCH, non-ketotic hyperglycemic chorea. The bold: with a P value < 0.05.

The collinearity analysis was conducted for the aforementioned independent variables. The results showed that irregular usage of glucose-lowering agents or no treatment, renal dysfunction, and HbA1c were included in the model. The analysis further revealed that worse renal function (eGFR <60 ml/min/1.73m2) [odds ratio (OR) 7.234, 95% confidence interval (CI) 1.885–27.756, *P*=0.004] and HbA1c level (OR 1.476, 95% CI 1.144–1.905, *P*=0.003) were significantly associated with NKHC (R2 = 39.7%) (**Supplementary Table 2**).

### Correlation analysis of resolution time of participants with NKHC

All patients with NKHC received strict glucose control after admission. Seventeen patients of NKHC required additional anti-epileptic/anti-psychotic agents. However, no significant correlations were found between the resolution time of chorea symptoms and any other variables, except for the use of additional anti-epileptic/anti-psychotic agents (r=0.535, *P*=0.015). The resolution time for participants requiring additional anti-epileptic/anti-psychotic medications was longer than for those who did not require these agents [21(15, 25) vs. 7(6, 10)].

## Discussion

To establish a diagnosis of NKHC, a comprehensive differential diagnosis must be conducted, considering various conditions such as vascular diseases, metabolic disorders (including acquired hepatolenticular degeneration and hyperthyroidism), infections (both bacterial and viral encephalitis), immune-mediated diseases, drug side effects (e.g., dopaminergic agents and tardive dyskinesia), carbon monoxide intoxication, and psychogenic chorea (4). Additionally, the presence of abnormalities on MRI/CT in NKHC must be differentiated from physiological and pathological calcification (e.g., Fahr’s disease and parathyroid dysfunction), Wilson disease, and carbon monoxide poisoning; such lesions typically manifest as bilateral or symmetrical abnormalities in the basal ganglia, whereas NKHC lesions tend to be unilateral (4). In this study, we carefully reviewed detailed medical records and confirmed the diagnosis of NKHC based on the criteria proposed by Chua et al. (8), after excluding the aforementioned conditions. Our main findings were that elevated HbA1c levels and renal dysfunction (eGFR <60 ml/min/1.73m2) were significantly associated with the development of NKHC.

Previous studies have postulated that a choreiform movement disorder may serve as an early indicator of diabetes (8). This study supports this hypothesis by revealing that a third of the patients with NKHC were diagnosed with diabetes for the first time during the study period. Therefore, patients with hyperglycemia associated with persistent, non-rhythmic, and unilateral or bilateral involuntary movements of their extremities should be suspected of having NKHC. Chorea may affect a wide range of body parts, including a single extremity, the upper and lower extremities unilaterally or bilaterally, the trunk, the neck and head, and also the face. One of the 20 participants with NKHC (No. 7) displayed bilateral involvement of their extremities, which is a relatively rare occurrence according to previous case reports (1, 8). Additionally, in this study, participants more frequently showed left-sided than right-sided lesions (13 cases *vs*. 5 cases, respectively), a rare occurrence.

We observed an association between renal dysfunction and NKHC. Diabetes mellitus is a leading cause of chronic kidney disease (CKD) and end-stage kidney disease (ESKD). Previous studies have shown that patients with diabetes and CKD frequently exhibit a choreiform movement disorder, particularly when undergoing dialysis (26–28). The glomerular filtration rate (GFR) is the best overall index of kidney function, estimated to assess the degree of kidney damage and monitor the progression of the disease (29). However, few studies have evaluated the relationship between estimated GFR (eGFR) and NKHC. In the present study, we found that the eGFRs of individuals with NKHC were significantly lower than those without NKHC. An eGFR <60 ml/min/1.73m^2^ is low, indicating impaired renal excretion (29). The resulting accumulation of toxic metabolites, such as urea, guanidines, and various amino acids, in the brain can lead to neuronal dysfunction through various mechanisms, including a neurotransmitter imbalance, an impairment in ATPase pump activity, calcium overload, and changes in plasma osmotic pressure (30–32). The renal impairment and subsequent decrease of eGFR in patients with NKHC may be caused by factors beyond diabetes alone, such as age, drug toxicity, and hypertension. As a cross-sectional study, it is not appropriate to establish a causal relationship between NKHC and renal dysfunction. However, no significant difference in demographic characteristics or comorbidity was found between individuals with NKHC and those without in our study. We believe that an eGFR <60 ml/min/1.73m^2^ may suggest metabolic disorders in the body that could accelerate the onset of NKHC. The specific pathogenesis of renal dysfunction in patients with NKHC requires further investigation, involving measures such as urinary albumin concentration, renal imaging, and renal biopsy.

The correlation between blood glucose level and the incidence of NKHC remains unconfirmed. The blood glucose concentration on admission (BGCA) of individuals with NKHC exhibited substantial variation, spanning a range from 5.9 mmol/L to 36.6 mmol/L. This variance may be attributed to the patients’ admission status, which could be influenced by factors such as food intake and the utilization of glucose-lowering agents. To minimize the impact of these potential confounding factors on the study findings, we employed a control group with BGCAs matched to those of the NKHC group.

The HbA1c values of individuals with NKHC (mean ± SD, 13 ± 2.82%) were significantly higher than those of the matched control group, with a previous systematic review and meta-analysis of 176 patients with diabetic striatopathy yielding a similar median HbA1c (13.1%) (8). A high HbA1c indicates elevated blood glucose concentrations over the preceding 2–3 months (33), suggesting that patients with poorly controlled diabetes during this period are more prone to developing NKHC. Previous studies have shown that the anaerobic pathway is used to provide energy for the basal ganglia during hyperglycemia (34), but this pathway is inefficient, especially in individuals with non-ketotic hyperglycemia rather than ketotic hyperglycemia. Therefore, basal ganglia dysfunction due to energy deficiency may be responsible for the development of chorea. This finding could potentially serve as a significant pointer towards a deeper comprehension of the intricate mechanisms behind NKHC.

In the present study, we observed that individuals with NKHC exhibited a higher frequency of basal ganglia lesions that appeared as hyperdensive lesions on CT (60%), hyperintensive lesions on T1-weighted MRI (100%), and hypointensive lesions on SWI (90.0%, n=11) (**Table 1**). While the precise etiology underlying these imaging characteristics remains elusive, pathological anatomy and MRI studies have suggested that these distinct signals may be attributed to reactive astrocytosis in the basal ganglia (7, 11, 35, 36). The hyperintensive signal on T1-weighted MRI could be attributed to the impact of oxidative stress on the hydration of cytoplasmic proteins in the swollen gamistocytes that characterize long-standing hyperglycemia. Furthermore, these astrocytes also express metallothionein, containing zinc, which is thought to be the cause of the hypointense signal on SWI (37). In our study, there were 11 participants with NKHC underwent SWI-MRI in the present study. Of these, four patients presented with unilateral chorea of the extremities, but had bilateral hypointensities in the basal ganglia on SWI. However, they did not have corresponding hyperintensive bilateral lesions of their basal ganglia on T1-weighted MRI, and only one of them had corresponding hyperdensive lesions on CT. It is noteworthy that these four participants were relatively old (84, 73, 84, and 83 years), and therefore these symmetrical signals (bilateral hypointensities in the basal ganglia on SWI-MRI) may represent age associated mineralization. Indeed, abnormal asymmetric lesions in the basal ganglia are responsible for NKHC (13). This study emphasizes the need for a multimodal MRI approach to assess NKHC.

In particular, we further investigated other MRI features of NKHC in patients. The middle cerebral artery supplies the basal ganglia, and there was no significant difference in the prevalence of middle cerebral artery stenosis between the participants with and without NKHC. The cells located in the periventricular region, deep white matter, and basal ganglia are particularly sensitive to ischemia (38). However, no severe PVHs or DWMHs were identified in the participants with NKHC. A previous study showed that hypoperfusion induced by hyperglycemia has a substantial effect on the transport of oxygen and nutrients, which results in cerebral degeneration and atrophy (39). However, the atrophy of the brains (indicated by both MTLA and VBR) of participants with NKHC did not differ from those without in the present study. The Rotterdam Scan Study (40) and a previous systematic review (41) suggested that there are close associations between epidemiological predictors of SBIs and diabetes mellitus. However, despite NKHC being a diabetes mellitus-related syndrome, the SBIs of the participants with NKHC were no worse than those of the non-NKHC group. This study adds to our understanding of the MRI features of NKHC, but further research is needed to fully understand its etiology and pathogenesis.

Our study found that resolution time of participants with additional anti-epileptic/anti-psychotic agents was longer than those without. The prognosis of chorea associated with nonketotic hyperglycemia has been reported as good generally. Chorea can be rapidly controlled by normalization of glycemia (42, 43). Resolution of the lesions seen in imaging studies was found to be slower than the clinical course (44). Previous study demonstrated abnormal imaging located on basal ganglia can persist for several months to years despite correction of hyperglycemia (1). Our study found that most patients with NKHC require additional anti-epileptic/anti-psychotic agents beside strict glucose control, which might be contributed to the prominently longer resolution time.

The present study’s strengths lie in its analytical approach, which, to the best of our knowledge, is the first of its kind for NKHC. Previous studies available to us were predominantly case reports and descriptive in nature. Additionally, we compared patients with NKHC with a matched control group and evaluated the correlation between NKHC and a comprehensive list of MRI variables (WMLs, VBR, MTLA, MCA-S, EPVS, and SBIs). However, this study also had some limitations. Firstly, the sample size was relatively small. Secondly, due to the retrospective nature of the study, we were unable to follow up with patients and assess whether neuroimaging abnormalities returned to normal following correction of hyperglycemia. Finally, despite our attempts to obtain imaging data prior to hospitalization or during follow up visits, it was often challenging to achieve this goal in a retrospective case-control study. A further prospective study dedicated to NKHC would help address these limitations.

## Conclusion

We discovered a significant independent association between patients with NKHC and higher HbA1c levels as well as renal dysfunction. These findings may contribute to the body of knowledge surrounding NKHC in the literature and offer a valuable clue for future investigations into its underlying mechanisms.

## Data availability statement

The raw data supporting the conclusions of this article will be made available by the authors, without undue reservation.

## Ethics statement

The studies involving humans were approved by Ethics Committee of the Tenth Affiliated Hospital of Southern Medical University (Dongguan People’s Hospital). The studies were conducted in accordance with the local legislation and institutional requirements. The ethics committee/institutional review board waived the requirement of written informed consent for participation from the participants or the participants’ legal guardians/next of kin because the retrospective design of the study and the minimal risk associated for the participants.

## Author contributions

Z-YL: Data curation, Formal analysis, Project administration, Writing – review & editing. Z-HL: Data curation, Formal analysis, Writing – review & editing. J-FQ: Conceptualization, Data curation, Formal analysis, Writing – original draft. Y-KC: Conceptualization, Supervision, Writing – review & editing.
