# Supplementary material for: Clinical and imaging associations for non-ketotic hyperglycemic chorea: a case-control study
Source: Front Endocrinol (Lausanne). 2023 Dec 20;14:1323942. doi: 10.3389/fendo.2023.1323942 (PMC10769489; doi:10.3389/fendo.2023.1323942)
Supplement: Supplementary file 1 [file Table_1.docx]

**Supplemental table 1. Comparison of the characteristics of the participants with NKHC and the patients that were excluded**

|  | NKHC | Hyperglycemia Chorea of Exclusion |  |  |
| --- | --- | --- | --- | --- |
|  | N=20 | N=7 | t/χ^2^/z-value | *P*-value |
| Age, years^*^ | 68.45±14.9 | 68.14±11.1 | 0.048 | 0.962 |
| Sex, Female^†^ | 9(45%) | 5 (71.4%) | 1.451 | 0.385 |
| Duration of DM, years^‡^ | 6(0, 17.5) | 13(8, 19) | -0.391 | 0.696 |
| BGCA, mmol/L^‡^ | 23.1(11.35, 30.58) | 23.5(19.9, 26.5) | -0.083 | 0.934 |
| HbA_1c_, %^*^ | 13±2.82 | 13.7±1.77 | -0.547 | 0.589 |

^*^ Mean (SD), *t*-test; ^†^ n (%), Fisher’s exact test; ^‡^ Median (interquartile range), Mann–Whitney U test. DM, diabetes mellitus; BGCA, blood glucose concentration on admission: NKHC, non-ketotic hyperglycemic chorea.

**Supplemental table 2. Results of the logistic regression of associated factors for non-ketotic hyperglycemic chorea**

|  | ***β*** | **OR (95% CI)** | ***P*-value** | **R^2^** |
| --- | --- | --- | --- | --- |
| Irregular glucose-lowering Drug Use or untreated | -0.954 | 0.385(0.106-1.402) | 0.148 |  |
| eGFR<60ml/min/1.73m^2^ | 1.979 | 7.234(1.885-27.756) | **0.004** |  |
| HbA_1c_ | 0.390 | 1.476(1.144-1.905) | **0.003** |  |
|  |  |  |  | **0.397** |

eGFR, Estimated Glomerular Filtration Rate.

**Supplemental table 3. Correlation analysis of resolution time of 20 participants with non-ketotic hyperglycemia chorea**

|  | **Correlation Coefficient** | ***P*-value** |
| --- | --- | --- |
| Age | -0.194 | 0.412 |
| Sex | -0.096 | 0.687 |
| Duration of DM | -0.249 | 0.291 |
| Irregular glucose-lowering Drug Use or untreated | -0.07 | 0.769 |
| No SMBG | 0.014 | 0.952 |
| BGCA | -0.103 | 0.665 |
| HbA1c | 0.183 | 0.44 |
| eGFR<60ml/min/1.73m | 0.113 | 0.634 |
| Additional anti-epileptic medications | 0.535 | 0.015 |

SMBG, self-monitoring of blood glucose; BGCA, blood glucose concentration on admission; eGFR, Estimated Glomerular Filtration Rate.
